# Efficacy and safety of single-inhaler extrafine triple therapy versus inhaled corticosteroid plus long-acting beta2 agonist in eastern Asian patients with COPD: the TRIVERSYTI randomised controlled trial

**DOI:** 10.1186/s12931-021-01683-2

**Published:** 2021-03-23

**Authors:** Jinping Zheng, Simonetta Baldi, Li Zhao, Huiping Li, Kwan-Ho Lee, Dave Singh, Alberto Papi, Frédérique Grapin, Alessandro Guasconi, George Georges

**Affiliations:** 1 State Key Laboratory of Respiratory Disease, National Clinical Research Centre for Respiratory Disease, Guangzhou Institute of Respiratory Health, First Affiliated Hospital of Guangzhou Medical University, Guangzhou, China; 2 Global Clinical Development, Chiesi Farmaceutici SpA, Largo Belloli, 11\a, 43122 Parma, Italy; 3 Shengjing Hospital of China Medical University, Shenyang, China; 4 Shanghai Pulmonary Hospital, Shanghai, China; 5 Yeungnam University Medical Center, Daegu, Republic of Korea; 6 Medicines Evaluation Unit, The University of Manchester, Manchester University NHS Foundations Trust, Manchester, UK; 7 Respiratory Medicine Unit, University of Ferrara, University Hospital S. Anna, Ferrara, Italy

**Keywords:** Triple inhalation therapy, Chronic obstructive pulmonary disease, Chronic bronchitis, Airway obstruction, Extrafine

## Abstract

**Background:**

A single-inhaler extrafine triple combination of beclometasone dipropionate (BDP), formoterol fumarate (FF) and glycopyrronium (G) has been developed for maintenance therapy of chronic obstructive pulmonary disease (COPD). This study evaluated the efficacy and safety of BDP/FF/G in patients in three eastern Asian areas: China, Republic of Korea and Taiwan.

**Methods:**

TRIVERSYTI was a double-blind, randomised, active-controlled, parallel-group study in patients with COPD, post-bronchodilator forced expiratory volume in 1 s (FEV_1_) < 50% predicted, ≥ 1 exacerbation in the previous 12 months, and receiving inhaled maintenance medication. Patients received either extrafine BDP/FF/G 100/6/10 µg via pressurised metered-dose inhaler, or non-extrafine budesonide/formoterol (BUD/FF) 160/4.5 µg via dry-powder inhaler, both administered as two puffs twice-daily for 24 weeks. The co-primary objectives (analysed in the overall population) were to demonstrate superiority of BDP/FF/G over BUD/FF for change from baseline in pre-dose morning and 2-h post-dose FEV_1_ at Week 24 (these were analysed as key secondary objectives in the China subgroup). The rate of moderate/severe COPD exacerbations was a secondary endpoint.

**Results:**

Of 708 patients randomised, 88.8% completed. BDP/FF/G was superior to BUD/FF for pre-dose and 2-h post-dose FEV_1_ at Week 24 [adjusted mean differences 62 (95% CI 38, 85) mL and 113 (87, 140) mL; both *p* < 0.001]. The annualised moderate/severe exacerbation rate was 43% lower with BDP/FF/G [rate ratio 0.57 (95% CI 0.42, 0.77); *p* < 0.001]. Adverse events were reported by 61.1% and 67.0% patients with BDP/FF/G and BUD/FF. Results were similar in the China subgroup.

**Conclusions:**

In patients with COPD, FEV_1_ < 50% and an exacerbation history despite maintenance therapy, treatment with extrafine BDP/FF/G improved bronchodilation, and was more effective at preventing moderate/severe COPD exacerbations than BUD/FF.

*Trial registration* CFDA CTR20160507 (registered 7 Nov 2016, http://www.chinadrugtrials.org.cn/index.html).

**Supplementary Information:**

The online version contains supplementary material available at 10.1186/s12931-021-01683-2.

## Background

Pharmacologic management of chronic obstructive pulmonary disease (COPD) aims to reduce current symptoms and future exacerbation risk [1]. ‘Triple therapy’ of an inhaled corticosteroid (ICS), a long-acting β_2_-agonist (LABA) and a long-acting muscarinic antagonist (LAMA) is an option for patients with COPD who exacerbate despite ICS/LABA or LABA/LAMA, or who experience symptoms with ICS/LABA [1]. Triple therapy can be administered as ICS/LABA in one inhaler and LAMA in a second, often with different design, instructions for use and dosing regimen; single-inhaler triple therapies simplify this regime, and potentially optimise adherence and persistence. One of these is extrafine beclometasone dipropionate (BDP), formoterol fumarate (FF) and glycopyrronium (G). Extrafine particles (i.e., mass median aerodynamic diameter < 2 µm) are more able to consistently reach small airways than non-extrafine particles [2], enhancing delivery to these airways, a major site of airflow obstruction in COPD [3, 4], with lower oropharyngeal deposition [5].

Three prior 52-week studies have evaluated BDP/FF/G in COPD: BDP/FF/G reduced the rate of COPD exacerbations by 23% versus BDP/FF in TRILOGY [6], by 20% versus tiotropium in TRINITY [7], and by 15% versus indacaterol/glycopyrronium in TRIBUTE [8]. All three studies were conducted in predominantly Caucasian populations. There is high prevalence of COPD in many countries in eastern Asia (13.6% in China [9]), so new therapeutic options are necessary in these countries. In addition, factors such as ethnicity, environment, education, nutrition and lifestyle may contribute to a differential response to pharmacotherapy. We therefore evaluated the efficacy and safety of BDP/FF/G in patients with COPD in three eastern Asian areas: China, Republic of Korea and Taiwan. To support regulatory approval of BDP/FF/G in China, the study was powered both for the overall population and for the subgroup of patients recruited at sites in China. Furthermore, although the primary analyses were for the overall population, the study’s key secondary objectives were analysed in the China subgroup, and all data are presented both overall and for the China subgroup.

## Methods

### Trial design

TRIVERSYTI was a multinational, double-blind, double-dummy, randomised, active-controlled, parallel-group study. Eligible patients were aged ≥ 40 years, diagnosed with COPD ≥ 12 months, current or ex-smokers (≥ 10 pack-years), post-bronchodilator forced expiratory volume in 1 s (FEV_1_) < 50% predicted, ≥ 1 exacerbation in the previous 12 months, and had been receiving therapy for ≥ 2 months with ICS/LABA, ICS/LAMA, LABA/LAMA, LAMA, or LABA (but not ICS/LABA/LAMA). Main exclusion criteria were: diagnosis of asthma; COPD exacerbation in the four weeks prior to entry; or use of traditional Chinese medicines for respiratory diseases. Patients provided written informed consent prior to any study-related procedure. Full inclusion and exclusion criteria are in the supplement.

After screening, eligible patients entered a two-week run-in receiving non-extrafine budesonide/formoterol (BUD/FF), 160/4.5 µg, two puffs twice daily (BID) via dry-powder inhaler. They were then randomised equally to extrafine BDP/FF/G 100/6/10 µg, two puffs BID via pressurised metered-dose inhaler, or to continue BUD/FF for 24 weeks. Patients were assigned to treatment centrally via interactive response technology (IRT), using a balanced block randomisation scheme stratified by area that was generated by the IRT provider. Patients, investigators, and site and sponsor staff were blinded to treatment assignment by a double-dummy design, with matching placebos.

Patients attended visits at randomisation (baseline) and after 4, 12, 18 and 24 weeks. Lung function [FEV_1_, forced vital capacity (FVC), forced mid-expiratory flow (FEF_25–75%_), and inspiratory capacity (IC)] were assessed pre-dose at each visit, with FEV_1_ and FVC also assessed at 2-h post-dose. The COPD Assessment Test (CAT) was administered at each visit, with the St George’s Respiratory Questionnaire (SGRQ) used at baseline and Weeks 12 and 24. COPD exacerbations were recorded throughout, with moderate exacerbations requiring treatment with systemic corticosteroids and/or antibiotics, and severe exacerbations resulting in hospitalisation or death. Salbutamol was permitted as rescue medication, with use recorded in diary cards. The occurrence of adverse events (AEs) was captured throughout, with safety evaluated by vital signs, electrocardiogram, haematology and blood chemistry.

The study was approved by independent ethics committees at each institution, and was performed in accordance with the principles of the Declaration of Helsinki, and the International Conference on Harmonisation notes for guidance on Good Clinical Practice (ICH/CPMP/135/95). Registered at the Chinese Food and Drug Administration (CFDA; registration number CTR20160507) and ClinicalTrials.gov (NCT03197818) websites.

### Outcomes

The co-primary objectives were to demonstrate superiority of BDP/FF/G over BUD/FF in the overall population for change from baseline in pre-dose morning and 2-h post-dose FEV_1_ at Week 24. To support regulatory approval of BDP/FF/G in China, the study’s key secondary objectives were to assess the co-primary objectives in the subgroup of patients recruited at sites in China (the China subgroup). Other secondary endpoints included: change from baseline in pre-dose and 2-h post-dose FEV_1_ at other visits and overall; FEV_1_ response (change from baseline ≥ 100 mL) at Week 24; change from baseline in pre-dose FVC, FEF_25–75%_, and IC (a measure of static hyperinflation), and 2-h post-dose FVC at all visits and overall; time to first moderate-or-severe COPD exacerbation; rate of moderate/severe COPD exacerbations; change from baseline in SGRQ total score at Weeks 12 and 24 and overall, and in CAT at all visits and overall; SGRQ response (decrease from baseline ≥ 4) at Week 24; change from baseline in rescue medication use in each inter-visit period and overall; and safety and tolerability.

### Sample size and statistical methods

The study intended to randomise a total of 990 patients. An interim analysis was planned when 614 randomised patients completed the study [with an estimated 499 (81%) in China]; assuming a non-evaluable rate of 13%, 534 would be evaluable at Week 24, providing approximately 89% power (80% for the China subgroup) to detect mean differences in favour of BDP/FF/G of 60 mL for pre-dose morning FEV_1_ and 70 mL for post-dose FEV_1_, assuming standard deviations of 200 mL for both. To avoid inflation of the Type I error, significance was adjusted using the Pocock-type error spending function method (considering a two-sided alpha of 0.0372). For the interim analysis, an independent data monitoring committee (DMC) reviewed unblinded data, with the DMC charter specifying the study would be stopped if BDP/FF/G was superior to BUD/FF for all co-primary and key secondary objectives. This was the case, and so further recruitment was stopped.

The co-primary endpoints were analysed using a linear mixed model for repeated measures including treatment, visit, treatment-by-visit interaction, area, COPD exacerbations in the previous year (1 or > 1), smoking status, and severity of airflow limitation (screening FEV_1_ < 30% or ≥ 30% predicted) as fixed effects, and baseline value and baseline-by-visit interaction as covariates. The co-primary and key secondary endpoints were analysed in hierarchical order: (1) pre-dose and (2) post-dose FEV_1_ in the overall intention-to-treat (ITT) population; (3) pre-dose and (4) post-dose FEV_1_ in the China subgroup. No superiority claim was possible unless the preceding test was significant in favour of BDP/FF/G.

The changes from baseline in FEV_1_, FVC, FEF_25–75%_, IC, SGRQ and CAT secondary endpoints were analysed using a similar model to the primary endpoints. A similar model was used to analyse the inter-visit rescue medication secondary endpoints, with inter-visit period used instead of visit in the model; the change from baseline for the entire treatment period was analysed using an analysis of covariance model including treatment, country, number of COPD exacerbations in the previous year and smoking status as fixed effects and the baseline value as a covariate. FEV_1_ response and SGRQ response were analysed using a logistic model including treatment, country, number of COPD exacerbations in the previous year and smoking status as factors and the baseline value as a covariate. The number of moderate/severe COPD exacerbations was analysed using a negative binomial model including treatment, country, number of COPD exacerbations in the previous year and smoking status as fixed effects, and log-time on study as an offset. The time to first COPD exacerbation was analysed using a Cox proportional hazards model including treatment, country, number of COPD exacerbations in the previous year and smoking status as factors.

Efficacy data were analysed in the ITT population, comprising all randomised patients who received at least one dose of study medication and with at least one post-baseline efficacy evaluation available. Safety analyses were performed in the safety population, which was all randomised patients who received at least one dose of study medication. The co-primary endpoints were also analysed in the per protocol (PP) population, which was all patients from the ITT population without any major protocol deviations. There was one protocol amendment after recruitment started, the main impact of which was to add the interim analysis. Data are presented overall and for the China subgroup.

## Results

### Participants

**Fig. 1:**
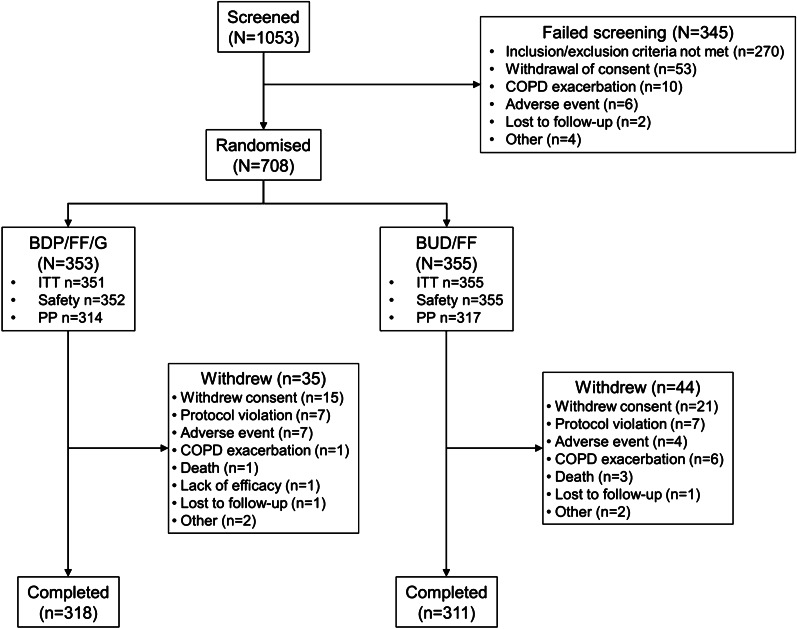
Patient flow through the study (overall population). *COPD* chronic obstructive pulmonary disease, *BDP* beclometasone dipropionate, *FF* formoterol fumarate, *G* glycopyrronium, *BUD* budesonide, *ITT* intention-to-treat, *PP* per protocol

The study was conducted in 63 centres (China 41, Republic of Korea 14, Taiwan 8) between 14 December 2016 and 26 May 2020. Of 708 patients randomised, 88.8% completed (90.1% and 87.6% in the BDP/FF/G and BUD/FF groups, respectively; Fig. 1). Disposition was similar for the China subgroup (Additional file 1: Figure S1). One patient in China did not receive study medication and was excluded from the BDP/FF/G safety and ITT populations; another patient in China had no post-baseline efficacy data, and was excluded from the BDP/FF/G ITT population. Baseline characteristics were similar between treatment groups, overall and in the China subgroup (Table 1).

**Table 1:** Patient baseline demographics and disease characteristics (ITT population)

	Overall population		China subgroup	
	BDP/FF/G (N = 351)	BUD/FF (N = 355)	BDP/FF/G (N = 286)	BUD/FF (N = 290)
*Areas, n (%)*				
China	286 (81.5)	290 (81.7)	286 (100)	290 (100)
Republic of Korea	54 (15.4)	54 (15.2)	–	–
Taiwan	11 (3.1)	11 (3.1)	–	–
Age, years	66.0 (7.1)	65.9 (7.7)	65.3 (7.1)	65.3 (7.4)
Males, n (%)	336 (95.7)	337 (94.9)	271 (94.8)	273 (94.1)
Body-mass index, kg/m^2^	21.8 (3.5)	21.3 (3.3)	21.8 (3.4)	21.5 (3.4)
*Smoking status, n (%)*				
Ex-smoker	266 (75.8)	266 (74.9)	215 (75.2)	215 (74.1)
Current smoker	85 (24.2)	89 (25.1)	71 (24.8)	75 (25.9)
Smoking history, pack-years	39.9 (24.0)	41.3 (22.5)	39.0 (23.9)	40.8 (23.0)
Time since first COPD diagnosis (years)	6.8 (5.9)	6.3 (4.9)	6.3 (5.9)	5.8 (4.6)
*FEV*_*1*_*, post-bronchodilator*				
Percent predicted	34.8 (8.9)	34.1 (8.6)	34.1 (8.9)	33.5 (8.7)
< 30% predicted, n (%)	113 (32.2)	119 (33.5)	103 (36.0)	106 (36.6)
30–50% predicted, n (%)	238 (67.8)	236 (66.5)	183 (64.0)	184 (63.4)
Reversibility in FEV_1_, %	12.7 (12.7)	12.5 (12.4)	13.8 (13.2)	13.0 (12.7)
CAT total score	16.7 (7.2)	17.4 (6.9)	16.6 (7.1)	17.3 (6.7)
SGRQ total score	44.1 (18.2)	43.3 (17.3)	43.9 (17.3)	43.1 (16.7)
*Exacerbations in previous year*, median (range)	1.0 (1, 11)	1.0 (1, 7)	1.0 (1, 11)	1.0 (1, 7)
1, n (%)	253 (72.1)	261 (73.5)	218 (76.2)	226 (77.9)
2, n (%)	58 (16.5)	62 (17.5)	41 (14.3)	43 (14.8)
≥ 3, n (%)	40 (11.4)	32 (9.0)	27 (9.4)	21 (7.2)
*COPD medication at study entry, n (%)*				
ICS/LABA	222 (63.2)	236 (66.5)	211 (73.8)	221 (76.2)
LABA/LAMA	46 (13.1)	51 (14.4)	2 (0.7)	9 (3.1)
LAMA	81 (23.1)	67 (18.9)	73 (25.5)	60 (20.7)
LABA	2 (0.6)	1 (0.3)	0	0

Data are mean (standard deviation) unless specified otherwise. *ITT* intention-to-treat, *BDP* beclometasone dipropionate, *FF* formoterol fumarate, *G* glycopyrronium, *BUD* budesonide, *COPD* chronic obstructive pulmonary disease, *FEV*_*1*_ forced expiratory volume in 1 s, *CAT* COPD assessment test, *SGRQ* St George’s Respiratory Questionnaire, *ICS* inhaled corticosteroid, *LABA* long-acting beta2-agonist, *LAMA* long-acting muscarinic antagonist

### Outcomes

#### Lung function

**Fig. 2:**
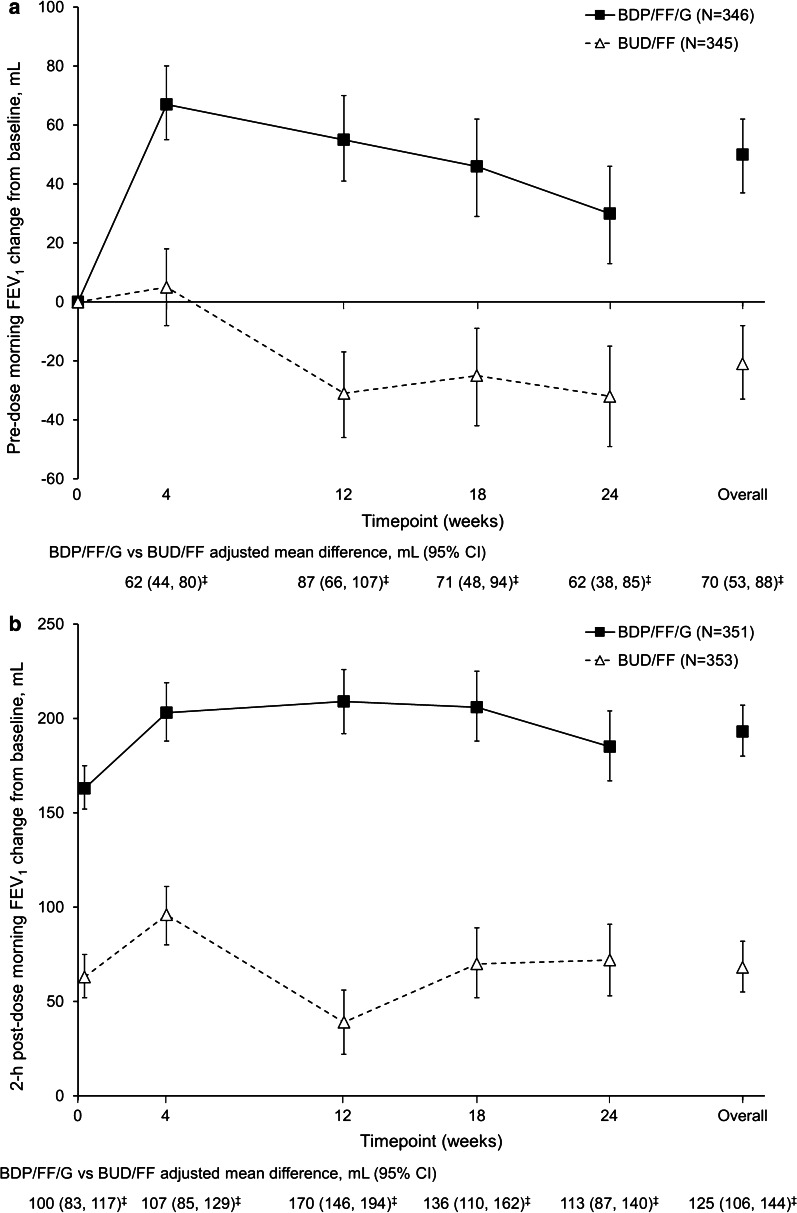
Adjusted mean change from baseline in **a** pre-dose morning FEV_1_, and **b** 2-h post-dose FEV_1_ (overall population, ITT). ^‡^*p* < 0.001. Data are adjusted mean and 95% CI. The N values are the number of patients included in the statistical model. *FEV*_*1*_ forced expiratory volume in 1 s, *ITT* intention-to-treat, *BDP* beclometasone dipropionate, *FF* formoterol fumarate, *G* glycopyrronium, *BUD* budesonide, *CI* confidence interval

In the overall population, for pre-dose and 2-h post-dose FEV_1_ at Week 24 BDP/FF/G was superior to BUD/FF, with adjusted mean differences [95% confidence interval (CI) ] of 62 (38, 85) mL and 113 (87, 140) mL, respectively (both *p* < 0.001). Results were similar in the China subgroup [63 (36, 90) mL and 120 (89, 151) mL, respectively; *p* < 0.001]. The results in the PP population were similar to those for the ITT population. Results at other visits were consistent with the Week 24 data, with significant BDP/FF/G versus BUD/FF differences in the overall population (Fig. 2a, b) and the China subgroup (Additional file 1: Figure S2).

**Fig. 3:**
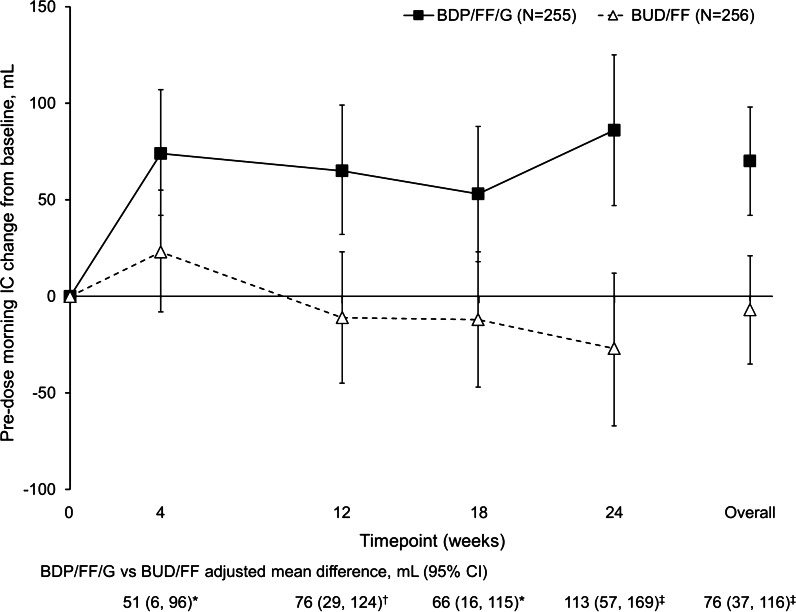
Adjusted mean change from baseline in IC (overall population, ITT). *p < 0.05; ^†^*p* < 0.01; ^‡^*p* < 0.001. Data are adjusted mean and 95% CI. The N values are the number of patients included in the statistical model. *IC* inspiratory capacity, *ITT* intention-to-treat, *BDP* beclometasone dipropionate, *FF* formoterol fumarate, *G* glycopyrronium, *BUD* budesonide, *CI* confidence interval

At Week 24, 91 (25.9%) patients had ≥ 100 mL change from baseline in pre-dose morning FEV_1_ in the BDP/FF/G group, compared with 43 (12.1%) in the BUD/FF group, with a significant odds ratio [OR; 2.58 (95% CI 1.72, 3.85); *p* < 0.001]. Results were similar in the China subgroup [82 (28.7%) and 39 (13.4%) patients, respectively; OR 2.59 (1.70, 3.97); *p* < 0.001]. FVC (pre-dose and 2-h post-dose) and FEF_25–75%_ (pre-dose) results were consistent with FEV_1_, with significant BDP/FF/G versus BUD/FF differences at all visits in the overall population and the China subgroup (Additional file 1: Figures S3–S5). There was a reduction in static lung hyperinflation in the BDP/FF/G group over the study (indicated by an increase from baseline in resting IC), with significant BDP/FF/G versus BUD/FF differences at all visits (Fig. 3; Additional file 1: Figure S6). This is important, as hyperinflation is one of the main reasons for activity limitation in patients with COPD, with reductions in hyperinflation associated with improvements in exercise tolerance [10–13].

#### Exacerbations

**Fig. 4:**
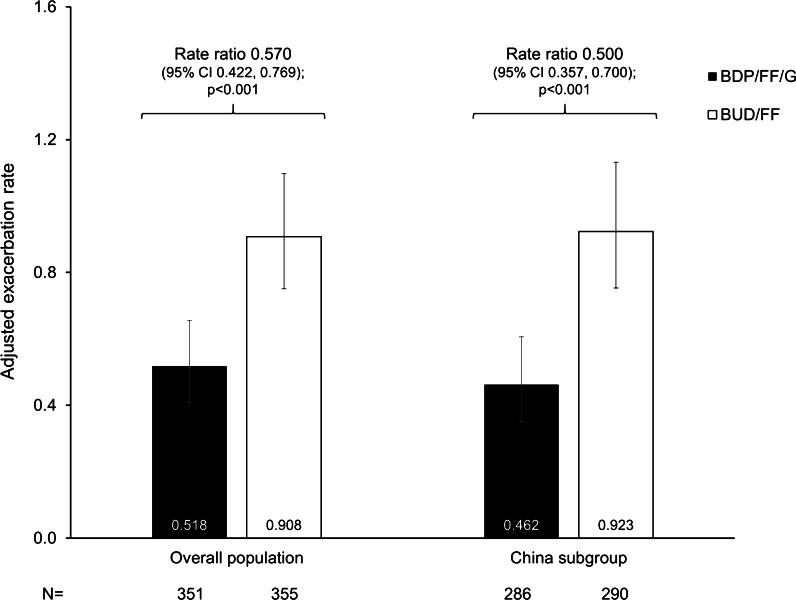
Annualised moderate/severe COPD exacerbation rates (ITT population). Bars represent adjusted exacerbation rate per patient per year and 95% CI. The N values are for the ITT population. *COPD* chronic obstructive pulmonary disease, *ITT* intention-to-treat, *BDP* beclometasone dipropionate, *FF* formoterol fumarate, *G* glycopyrronium, *BUD* budesonide, *CI* confidence interval

The annualised moderate/severe exacerbation rate was 43% lower overall with BDP/FF/G than BUD/FF, with a 50% reduction in the China subgroup (*p* < 0.001; Fig. 4). In the overall population, 66 (18.8%) patients in the BDP/FF/G group had ≥ 1 moderate/severe COPD exacerbation, versus 109 (30.7%) with BUD/FF; the time to first moderate-or-severe exacerbation significantly extended with BDP/FF/G [hazard ratio 0.55 (95% CI 0.40, 0.75); *p* < 0.001; Additional file 1: Figure S7A]. In the China subgroup, 49 (17.1%) and 92 (31.7%) patients, respectively, had an exacerbation, with the time to first moderate-or-severe COPD exacerbation again significantly longer in the BDP/FF/G group [0.48 (95% CI 0.34, 0.68); *p* < 0.001; Additional file 1: Figure S7B].

#### Health status

**Fig. 5:**
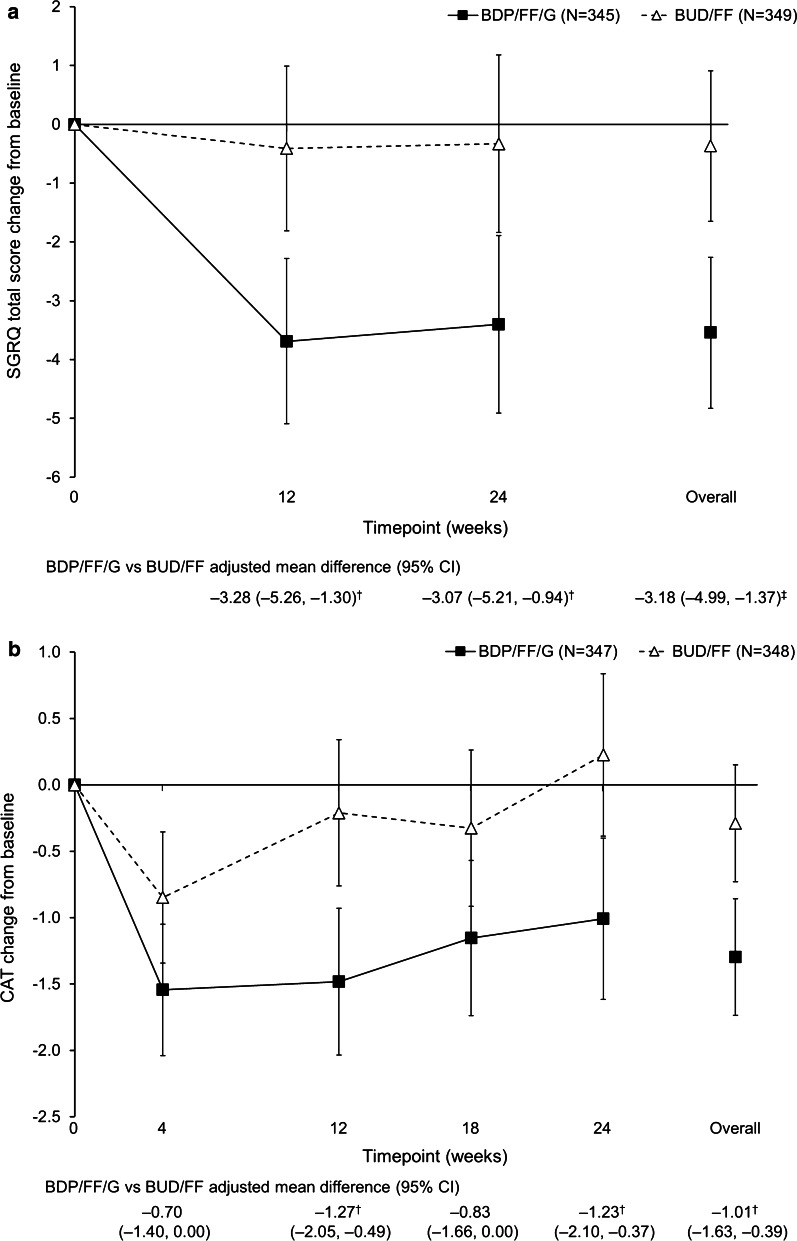
Adjusted mean change from baseline in **a** SGRQ and **b** CAT total scores (overall population, ITT). ^†^*p* < 0.01; ^‡^*p* < 0.001. Data are adjusted mean and 95% CI. The N values are the number of patients included in the statistical model. *SGRQ* St George’s Respiratory Questionnaire, *CAT* COPD Assessment Test, *ITT* intention-to-treat, *BDP* beclometasone dipropionate, *FF* formoterol fumarate, *G* glycopyrronium, *BUD* budesonide, *CI* confidence interval

SGRQ and CAT total scores improved (scores decreased) from baseline with BDP/FF/G, but were unchanged with BUD/FF; differences between groups were significant in both SGRQ assessments, and at Weeks 12 and 24 (and also at Week 4 in the China subgroup) for the CAT assessments (Fig. 5; Additional file 1: Figure S8). At Week 24, 149 patients (42.5%) overall were SGRQ responders (≥ 4 unit decrease from baseline) with BDP/FF/G, compared to 119 (33.5%) with BUD/FF [OR 1.48 (95% CI 1.07, 2.05); *p* = 0.018]. In the China subgroup, 127 (44.4%) and 97 (33.4%) patients, respectively, were responders [1.61 (1.12, 2.30; *p* = 0.010)].

#### Rescue medication use

Rescue medication use decreased from baseline in both groups. The decrease with BDP/FF/G was larger, although differences between groups were not consistently significant (Additional File 1: Figures S9 and S10).

### Safety

Fewer patients experienced AEs with BDP/FF/G than BUD/FF, predominantly due to fewer COPD exacerbations (Table 2). The majority of AEs were mild or moderate, with few study-related. The only treatment-related serious AE was liver injury, in a patient in the China subgroup receiving BDP/FF/G; this patient subsequently recovered. Four patients died during the study, all in the China subgroup, none study-related. One patient was receiving BDP/FF/G (the AE was recorded only as death). The only AE leading to study-drug discontinuation in more than one patient with either treatment was COPD exacerbation: One patient with BDP/FF/G and seven with BUD/FF. Fewer patients had pneumonia AEs with BDP/FF/G than BUD/FF, both overall (2.3% versus 3.7%) and in the China subgroup (1.4% versus 2.4%).

**Table 2:** Adverse events and serious adverse events (safety population)

Number (%) of patients	Overall population		China subgroup	
	BDP/FF/G (N = 352)	BUD/FF (N = 355)	BDP/FF/G (N = 287)	BUD/FF (N = 290)
*Adverse events*	215 (61.1)	238 (67.0)	173 (60.3)	195 (67.2)
COPD exacerbation	66 (18.8)	110 (31.0)	49 (17.1)	93 (32.1)
Upper respiratory tract infection	58 (16.5)	49 (13.8)	55 (19.2)	45 (15.5)
Nasopharyngitis	22 (6.3)	23 (6.5)	16 (5.6)	17 (5.9)
Hypertension	12 (3.4)	24 (6.8)	10 (3.5)	19 (6.6)
Productive cough	9 (2.6)	9 (2.5)	8 (2.8)	8 (2.8)
Pneumonia	8 (2.3)	13 (3.7)	4 (1.4)	7 (2.4)
Cough	8 (2.3)	8 (2.3)	4 (1.4)	7 (2.4)
Bronchitis	6 (1.7)	3 (0.8)	5 (1.7)	1 (0.3)
Pharyngitis	5 (1.4)	3 (0.8)	5 (1.7)	3 (1.0)
Dyspnoea	4 (1.1)	8 (2.3)	3 (1.0)	5 (1.7)
Gamma-glutamyltransferase increased	2 (0.6)	6 (1.7)	2 (0.7)	4 (1.4)
Hepatic function abnormal	2 (0.6)	6 (1.7)	2 (0.7)	6 (2.1)
Alanine aminotransferase increased	2 (0.6)	6 (1.7)	1 (0.3)	3 (1.0)
Back pain	0	8 (2.3)	0	8 (2.8)
Hypokalaemia	0	7 (2.0)	0	5 (1.7)
Neutrophil count increased	6 (1.7)	2 (0.6)	6 (2.1)	1 (0.3)
Anaemia	4 (1.1)	5 (1.4)	4 (1.4)	5 (1.7)
Lung infection	2 (0.6)	5 (1.4)	2 (0.7)	5 (1.7)
Blood pressure increased	1 (0.3)	5 (1.4)	1 (0.3)	5 (1.7)
*Serious adverse events*	40 (11.4)	60 (16.9)	30 (10.5)	49 (16.9)
COPD exacerbation	20 (5.7)	43 (12.1)	16 (5.6)	38 (13.1)
Pneumonia	4 (1.1)	9 (2.5)	1 (0.3)	4 (1.4)
Pneumothorax spontaneous	2 (0.6)	0	2 (0.7)	0
Influenza	2 (0.6)	0	0	0
*Treatment-related adverse events*	9 (2.6)	16 (4.5)	8 (2.8)	14 (4.8)
Muscle spasms	2 (0.6)	2 (0.6)	2 (0.7)	2 (0.7)
Blood pressure increased	0	2 (0.6)	0	2 (0.7)
*Treatment-related serious adverse events*	1 (0.3)	0	1 (0.3)	0
*Severe adverse events*	31 (8.8)	56 (15.8)	23 (8.0)	48 (16.6)
COPD exacerbation	22 (6.3)	46 (13.0)	18 (6.3)	41 (14.1)
Pneumonia	3 (0.9)	8 (2.3)	2 (0.7)	4 (1.4)
Influenza	2 (0.6)	0	0	0
Lung infection	1 (0.3)	2 (0.6)	1 (0.3)	2 (0.7)
*Adverse events leading to study drug discontinuation*	8 (2.3)	13 (3.7)	8 (2.8)	10 (3.4)
COPD exacerbation	1 (0.3)	7 (2.0)	1 (0.3)	5 (1.7)
*Adverse events leading to death*	1 (0.3)	3 (0.8)	1 (0.3)	3 (1.0)

Data are n (%). ≥ 1.5% in either group of the overall population for adverse events and ≥ 0.5% in either group of the overall population for serious adverse events, treatment-related adverse events, and adverse events leading to study drug discontinuation. *BDP* beclometasone dipropionate, *FF* formoterol fumarate, *G* glycopyrronium, *BUD* budesonide, *COPD* chronic obstructive pulmonary disease

There were few clinically relevant changes in haematology or biochemistry parameters, and minimal changes in any vital sign parameters. The incidence of clinically relevant QTc interval values was low and not different between treatments; no males had QTc interval values > 480 ms, and no females had values > 500 ms, and there were no changes > 60 ms.

## Discussion

In this study, triple therapy with extrafine BDP/FF/G improved lung function and health status compared with non-extrafine BUD/FF, with significant reductions in the incidence and rate of COPD exacerbations. The results in the China subgroup were consistent with the overall population, with BDP/FF/G demonstrating similar efficacy to the overall population and a good overall safety profile.

Our results are consistent with those of a number of previous 52-week triple therapy versus ICS/LABA studies [6, 14–16], although we specifically recruited only patients from eastern Asia. In particular, the results of the TRILOGY study, in which differences at Week 26 between BDP/FF/G and BDP/FF were 81 and 117 mL for pre-dose and 2-h post-dose FEV_1_, respectively [6], were similar to those observed here (62 mL and 113 mL, respectively). The TRIVERSYTI study design specified that recruitment should be stopped after 614 randomised patients were evaluated if statistically significant outcomes were observed for the co-primary and key secondary objectives. Although there would be no overt harm in continuing to recruit patients into the study, the effect of BDP/FF/G on these objectives was sufficiently marked (and positive) that the independent DMC recommended that no further subject recruitment into TRIVERSYTI was necessary.

While the TRIVERSYTI study primary analysis was focused on lung function endpoints, the enrolled patients were required to have had at least one exacerbation in the previous year, to recruit patients at increased exacerbation risk, such that treatment differences on exacerbation rate could be properly evaluated. The annualised moderate/severe exacerbation rate in the ICS/LABA group in TRIVERSYTI (0.91) was higher than in TRILOGY (0.56), as was the rate reduction (43% versus 23%). The large effect size for such a modest sample size suggests that the population recruited into TRIVERSYTI may gain even more benefit from treatment—although the ICS/LABAs differed in the two studies, with extrafine BDP/FF used in TRILOGY and non-extrafine BUD/FF in TRIVERSYTI. Importantly, the improved efficacy of BDP/FF/G versus BUD/FF was accompanied by an overall good safety profile—and in particular with a lower incidence of pneumonia. This lower pneumonia incidence could be due to differences in the ICS, the dose or formulation (extrafine BDP, compared to non-extrafine BUD); however, given the addition of the LAMA to ICS/LABA reduces the incidence of exacerbations, one may expect this reduced pneumonia risk from BDP/FF/G.

To our knowledge, this is the first study specifically designed to evaluate the efficacy of single-inhaler triple therapy in patients from eastern Asia. One previous study evaluated the efficacy of tiotropium plus BUD/FF in five eastern Asian areas, in which triple therapy improved bronchodilation and health status compared with tiotropium alone, with a 40.7% reduction in exacerbation rate (of note, patients in the tiotropium group were not permitted ICS) [17]. Subgroup analyses in patients from China have been published from two other single-inhaler triple therapy studies: in FULFIL, triple therapy with fluticasone furoate/umeclidinium/vilanterol improved bronchodilation compared with BUD/FF, with numerical improvements in health status and exacerbation rate [18], whereas in KRONOS BUD/glycopyrrolate/FF improved bronchodilation and health status versus BUD/FF, with a non-statistically significant 49% reduction in moderate/severe exacerbation rate [19]. Our results are broadly consistent with these, but with a much larger sample size, and with a statistically significant reduction in the rate of moderate/severe exacerbations.

We recognise that TRIVERSYTI has some limitations. Unlike in TRILOGY, the comparator in TRIVERSYTI differed in terms of molecules, formulation and device (when TRIVERSYTI was initiated, BDP/FF was not approved for COPD in China, Republic of Korea or Taiwan). It is unclear, therefore, which characteristic (or characteristics) was responsible for the observed improved efficacy (although the inclusion of the LAMA component in BDP/FF/G is likely responsible for much of the difference, as we observed in TRILOGY [6]). This reflects standard clinical practice, however, in that treatment choices are based not only on molecules but also on inhaler devices. In addition, more than 80% of patients were recruited by sites in China, and so the results of the overall population are largely driven by the China subgroup.

## Conclusions

In conclusion, this study is the first to evaluate single-inhaler triple therapy in patients recruited solely in eastern Asia. In patients with COPD, FEV_1_ < 50% and an exacerbation history despite maintenance therapy, treatment with extrafine BDP/FF/G improved bronchodilation and health status, and was more effective at preventing moderate/severe COPD exacerbations than BUD/FF.

## Supplementary Information

**Additional file 1.** Supplementary methods and results supporting the main body of the manuscript.

## Data Availability

Chiesi commits to sharing with qualified scientific and medical Researchers, conducting legitimate research, patient-level data**,** study-level data, the clinical protocol and the full clinical study report of Chiesi Farmaceutici SpA-sponsored interventional clinical trials in patients for medicines and indications approved by the European Medicines Agency and/or the US Food and Drug Administration after 1st January 2015, following the approval of any received research proposal and the signature of a Data Sharing Agreement. Chiesi provides access to clinical trial information consistently with the principle of safeguarding commercially confidential information and patient privacy. Other information on Chiesi’s data sharing commitment, access and research request’s approval process are available in the Clinical Trial Transparency section of http://www.chiesi.com/en/research-and-development/.

## Abbreviations

AE: Adverse event
BDP: Beclometasone dipropionate
BID: Twice daily
BUD: Budesonide
CAT: COPD Assessment Test
CI: Confidence interval
COPD: Chronic obstructive pulmonary disease
DMC: Data monitoring committee
FEF_25–75%_: Forced mid-expiratory flow
FEV_1_: Forced expiratory volume in 1 s
FF: Formoterol fumarate
FVC: Forced vital capacity
G: Glycopyrronium
IC: Inspiratory capacity
ICS: Inhaled corticosteroid
IRT: Interactive response technology
ITT: Intention-to-treat
LABA: Long-acting β_2_-agonist
LAMA: Long-acting muscarinic antagonist
OR: Odds ratio
PP: Per protocol
SGRQ: St George’s Respiratory Questionnaire
