# Supplementary material for: Efficacy and safety of single-inhaler extrafine triple therapy versus inhaled corticosteroid plus long-acting beta2 agonist in eastern Asian patients with COPD: the TRIVERSYTI randomised controlled trial
Source: Respir Res. 2021 Mar 23;22:90. doi: 10.1186/s12931-021-01683-2 (PMC7989027; doi:10.1186/s12931-021-01683-2)
Supplement: Supplementary file 1 — Additional file 1. Supplementary methods and results supporting the main body of the manuscript. [file 12931_2021_1683_MOESM1_ESM.docx]

# Efficacy and safety of single-inhaler extrafine triple therapy versus inhaled corticosteroid plus long-acting beta2 agonist in eastern Asian patients with COPD: The TRIVERSYTI randomised controlled trial

Jinping Zheng, Simonetta Baldi, Li Zhao, Huiping Li, Kwan-Ho Lee, Dave Singh, Alberto Papi, Frédérique Grapin, Alessandro Guasconi, George Georges.

# Additional file 1

## Methods

### Inclusion criteria

Patients must have met all of the following inclusion criteria to be eligible for enrolment into the study:

1. Male and female adults aged ≥40 years with written informed consent obtained prior to any study-related procedure.

2. Patients with a diagnosis of COPD (according to the Global Initiative for Chronic Obstructive Lung Disease strategic document, updated January 2015) at least 12 months before the screening visit.

3. A smoking history of at least 10 pack years [pack-years = (number of cigarettes per day x number of years)/20]. Current and ex-smokers were eligible. Smoking cessation therapy must have been completed six months prior to screening visit.

4. A post-bronchodilator FEV_1_ <50% of the predicted normal value and a postbronchodilator FEV_1_/FVC ratio < 0.7 at least 10-15 min after 4 puffs (4 x 100 mcg) of salbutamol pressurised metered-dose inhaler. If this criterion was not met at screening, the test could be repeated once before randomisation visit.

5. A documented history of at least one exacerbation in the 12 months preceding the screening visit, with COPD exacerbations defined as:

*“A sustained worsening of the patient’s condition (dyspnoea, cough and/or sputum production/purulence), from the stable state and beyond normal day-to-day variations, that is acute in onset and necessitates a change in regular medication in a patient with underlying COPD that includes prescriptions of systemic corticosteroids and/or antibiotics or need for hospitalisation”*

6. Patients receiving therapy for at least two months prior to screening with:

- Inhaled corticosteroid/long-acting β_2_-agonist or

- Inhaled corticosteroid/long-acting muscarinic antagonist or

- Inhaled long-acting β_2_-agonist and inhaled long-acting muscarinic antagonist or

- Long-acting muscarinic antagonist or

- Long-acting β_2_-agonist

7. A cooperative attitude and ability to be trained to use correctly the study inhalers (pressurised metered-dose inhaler and dry-powder inhaler).

8. A cooperative attitude and ability to be trained to use correctly the COPD questionnaires.

At screening visit (Visit 1), all inclusion criteria were checked.

At the randomisation visit (Visit 2), the following criteria were re-checked: 7, 8

### Exclusion criteria

The presence of any of the following excluded a patient from study enrolment:

1. Pregnant or lactating women and all women physiologically capable of becoming pregnant (i.e. women of childbearing potential) UNLESS they were willing to use one or more of the following reliable methods of contraception:

a. Placement of an intrauterine device or intrauterine system.

b. Hormonal contraception (implantable, patch, oral).

c. Barrier methods of contraception: condom or occlusive cap (diaphragm or cervical vaults/caps) with spermicidal foam/gel/film/cream/suppository.

d. Male sterilisation (with the appropriate post-vasectomy documentation of the absence of sperm in the ejaculate).

Reliable contraception was to be maintained throughout the study until last study visit. “True abstinence” was acceptable only if it was in line with the preferred and usual lifestyle of the patient. Any postmenopausal women (physiologic menopause defined as 12 consecutive months of amenorrhoea) or women permanently sterilised (e.g., tubal occlusion, hysterectomy or bilateral salpingectomy) could be enrolled in the study.

2. Diagnosis of asthma, history of allergic rhinitis or atopy (atopy which may raise contraindications or impact the efficacy of the study according to Investigator’s judgment).

3. Patients requiring use of the following medications:

a. Systemic steroids for COPD exacerbation in the four weeks prior to screening.

b. A course of antibiotics for COPD exacerbation longer than seven days in the four weeks prior to screening.

c. Phosphodiesterase inhibitors in the four weeks prior to screening.

d. Use of antibiotics for a lower respiratory tract infection (e.g. pneumonia) in the four weeks prior to screening.

4. COPD exacerbation requiring prescriptions of systemic corticosteroids and/or antibiotics or hospitalisation during the run-in period.

5. Changes in dose, schedule, formulation or product of oral xanthine derivatives (e.g., theophylline) in the month prior to screening visit or during the run-in period. Stopping of xanthines prior to the screening visit was allowed.

6. Patients treated with non-cardioselective β-blockers in the week preceding the screening visit or during the run-in period.

7. Patients treated with long-acting antihistamines (e.g. astemizole, terfenadine), unless taken at stable regimen for at least two months prior to screening for the duration of the study, or if taken when required.

8. Patients requiring long term (at least 12 hours daily) oxygen therapy for chronic hypoxaemia.

9. Known respiratory disorders other than COPD which may impact the efficacy of the study drug according the investigator’s judgment. This could include but was not limited to α-1 antitrypsin deficiency, active tuberculosis, lung cancer, bronchiectasis, sarcoidosis, lung fibrosis, pulmonary hypertension and interstitial lung disease.

10. Patients who had a clinically significant cardiovascular condition (such as but not limited to unstable ischemic heart disease, New York Heart Association Class III/IV, left ventricular failure, acute myocardial infarction), advanced atrio-ventricular conduction blocks.

11. Patients with atrial fibrillation:

- Paroxysmal (i.e., intermittent).
- Persistent as defined by continuous atrial fibrillation diagnosed for less than six months.
- Persistent for at least six months with a resting ventricular rate ≥100/min controlled with a rate control strategy (i.e. selective β-blocker, calcium channel blocker, pacemaker placement, digoxin or ablation therapy).

12. An abnormal and clinically significant 12-lead electrocardiogram (ECG) that resulted in an active medical problem that may impact the safety of the patient according to Investigator’s judgement. Patients whose 12 lead ECG shows QTcF >450 ms for males or QTcF >470 ms for females at screening and at randomisation visits were not eligible.

13. Medical diagnosis of narrow-angle glaucoma, clinically relevant prostatic hypertrophy or bladder neck obstruction that in the opinion of the Investigator would prevent use of anticholinergic agents.

14. History of hypersensitivity to M3 antagonists, β2-agonists, corticosteroids or any of the excipients contained in any of the formulations used in the trial.

15. Clinically significant laboratory abnormalities indicating a significant or unstable concomitant disease which may impact the efficacy or the safety of the study drug according to Investigator’s judgement.

16. Patients with serum potassium levels <3.5 mEq/L (or 3.5 mmol/L) at screening.

17. Unstable concurrent disease: e.g. uncontrolled hyperthyroidism, uncontrolled diabetes mellitus or other endocrine disease; uncontrolled gastrointestinal disease (e.g. active peptic ulcer); neurological disease; uncontrolled haematological disease; uncontrolled autoimmune disorders, or other which may impact the feasibility of the results of the study according to Investigator’s judgment.

18. History of alcohol abuse and/or substance/drug abuse within 12 months prior to screening visit.

19. Participation in another clinical trial where investigation drug was received less than eight weeks prior to screening visit.

20. Patients treated with Traditional Chinese Medicines used for respiratory diseases.

At the screening visit (Visit 1), all exclusion criteria were checked except criterion 4. At the randomisation visit (Visit 2), the following criteria were re-checked: 1 ,4 , 5, 6, 7, 10, 11, 12, 17, 20.

An additional exclusion criterion was applied at sites in Republic of Korea only: History of hypertrophic cardiomyopathy.

## Results

Supplementary Figure 1. Patient flow through the study (China subgroup)


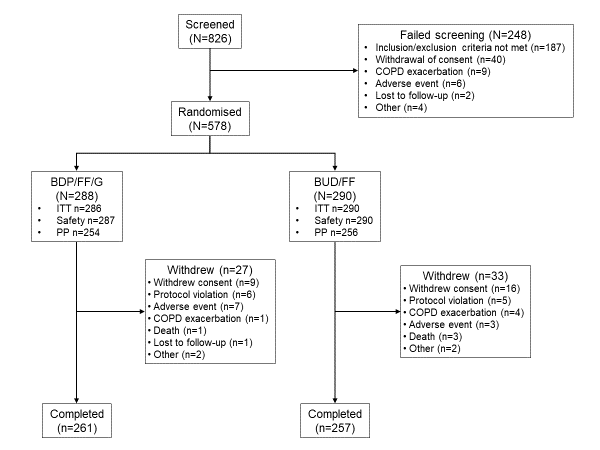


Abbreviations: COPD, chronic obstructive pulmonary disease; BDP, beclometasone dipropionate; FF, formoterol fumarate; G, glycopyrronium; BUD, budesonide; ITT, intention-to-treat; PP, per protocol.

Supplementary Figure 2. Adjusted mean change from baseline in A) pre-dose morning FEV_1_ and B) 2-h post-dose FEV_1_ (China subgroup, ITT)


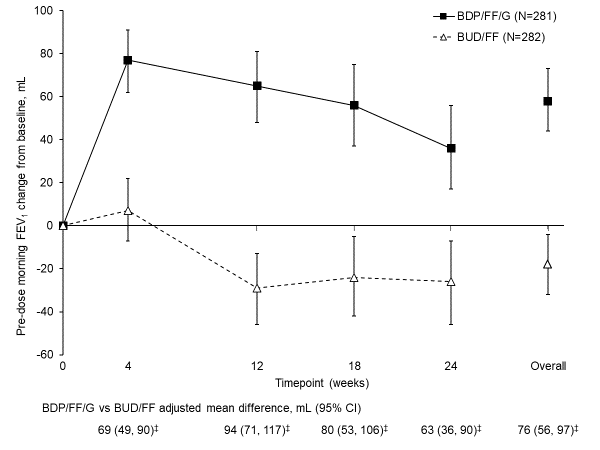


**A)**


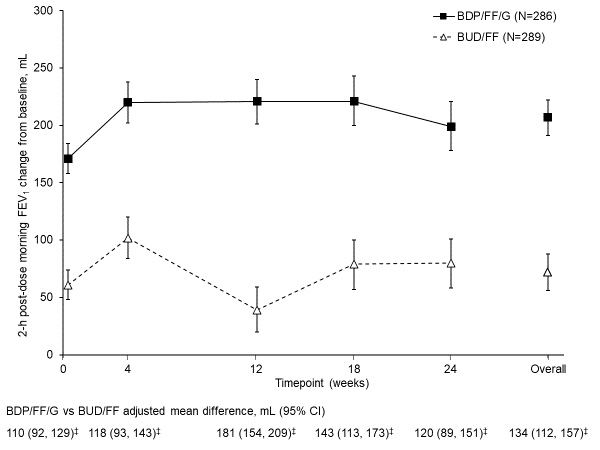


**B)**

^‡^p<0.001. Data are adjusted mean and 95% CI. The N values are the number of patients included in the statistical model. Abbreviations: FEV_1_, forced expiratory volume in 1 second; ITT, intention-to-treat; BDP, beclometasone dipropionate; FF, formoterol fumarate; G, glycopyrronium; BUD, budesonide; CI, confidence interval.

Supplementary Figure 3. Adjusted mean change from baseline in pre-dose morning FVC, in A) the overall population and B) the China subgroup (ITT)


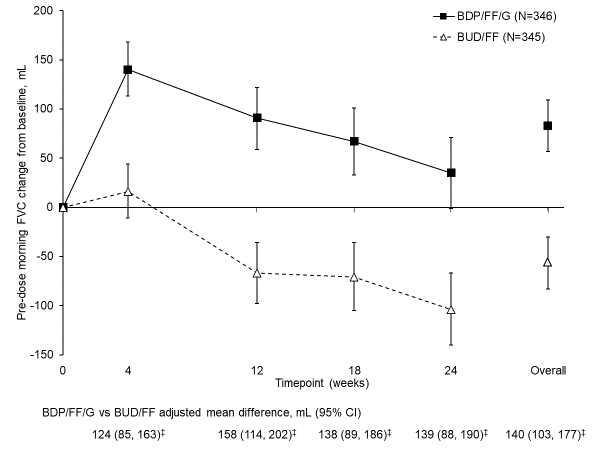


**A)**


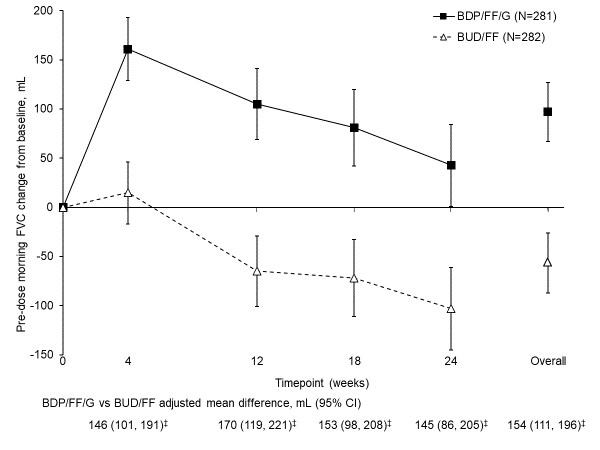


**B)**

^‡^p<0.001. Data are adjusted mean and 95% CI. The N values are the number of patients included in the statistical model. Abbreviations: FVC, forced vital capacity; ITT, intention-to-treat; BDP, beclometasone dipropionate; FF, formoterol fumarate; G, glycopyrronium; BUD, budesonide; CI, confidence interval.

Supplementary Figure 4. Adjusted mean change from baseline in 2-h post-dose FVC, in A) the overall population and B) the China subgroup (ITT)


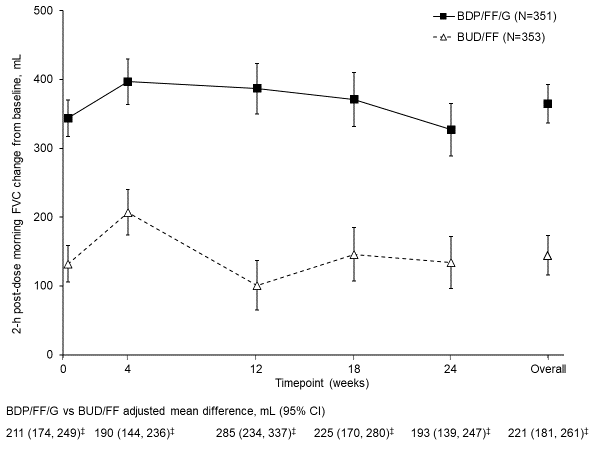


**A)**


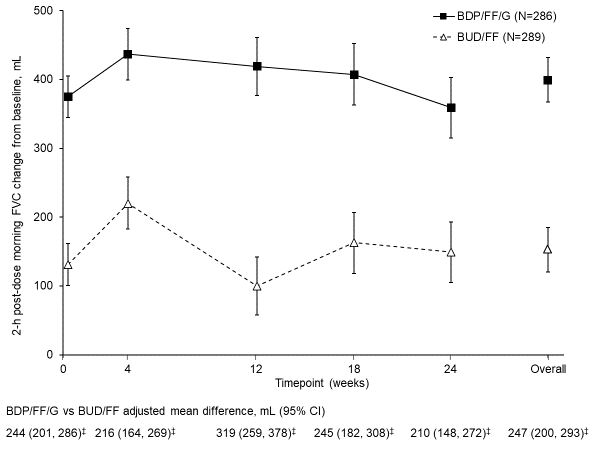


**B)**

^‡^p<0.001. Data are adjusted mean and 95% CI. The N values are the number of patients included in the statistical model. Abbreviations: FVC, forced vital capacity; ITT, intention-to-treat; BDP, beclometasone dipropionate; FF, formoterol fumarate; G, glycopyrronium; BUD, budesonide; CI, confidence interval.

Supplementary Figure 5. Adjusted mean change from baseline in pre-dose morning
FEF_25–75%_, in A) the overall population and B) the China subgroup (ITT)


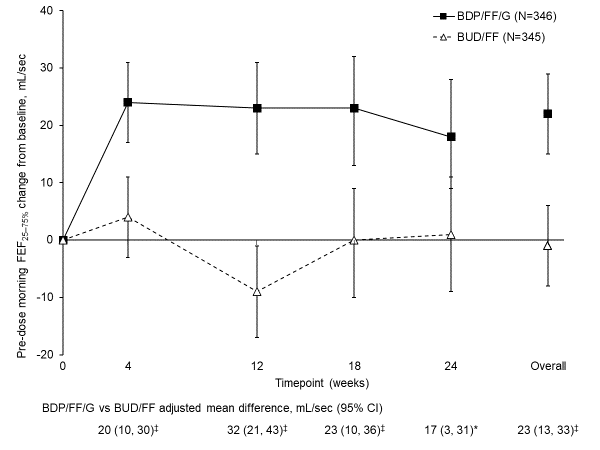


**A)**


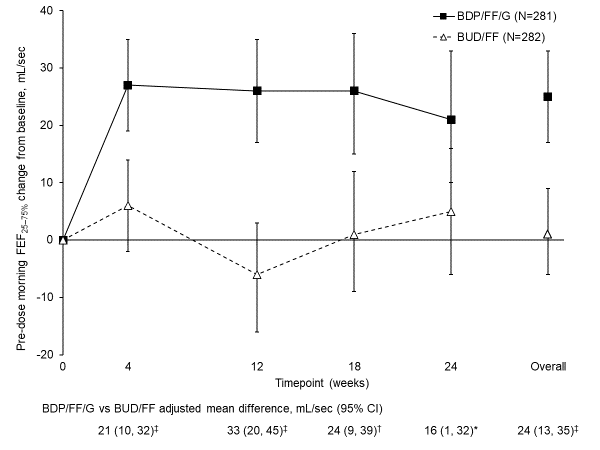


**B)**

*p<0.05, ^†^p<0.01, ^‡^p<0.001. Data are adjusted mean and 95% CI. The N values are the number of patients included in the statistical model. Abbreviations: FEF_25–75%_, forced mid-expiratory flow; ITT, intention-to-treat; BDP, beclometasone dipropionate; FF, formoterol fumarate; G, glycopyrronium; BUD, budesonide; CI, confidence interval.

Supplementary Figure 6. Adjusted mean change from baseline in pre-dose morning IC (China subgroup, ITT)


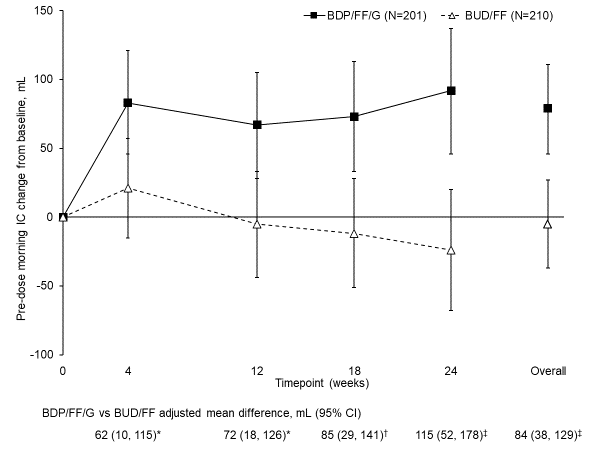


*p<0.05, ^†^p<0.01, ^‡^p<0.001. Data are adjusted mean and 95% CI. The N values are the number of patients included in the statistical model. Abbreviations: IC, inspiratory capacity; ITT, intention-to-treat; BDP, beclometasone dipropionate; FF, formoterol fumarate; G, glycopyrronium; BUD, budesonide; CI, confidence interval.

Supplementary Figure 7. Time to first moderate/severe COPD exacerbation in A) the overall population and B) the China subgroup (ITT)


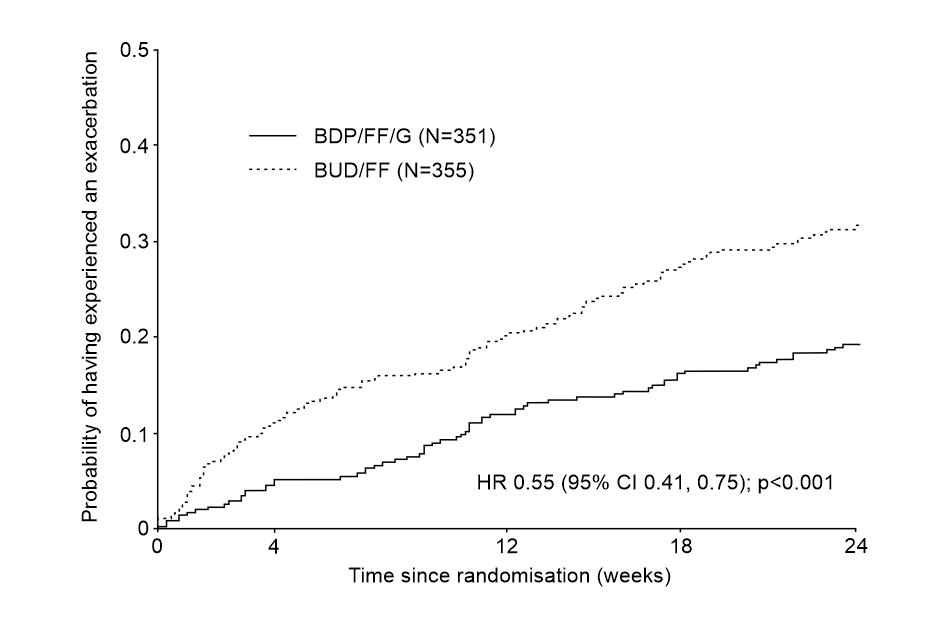


**A)**


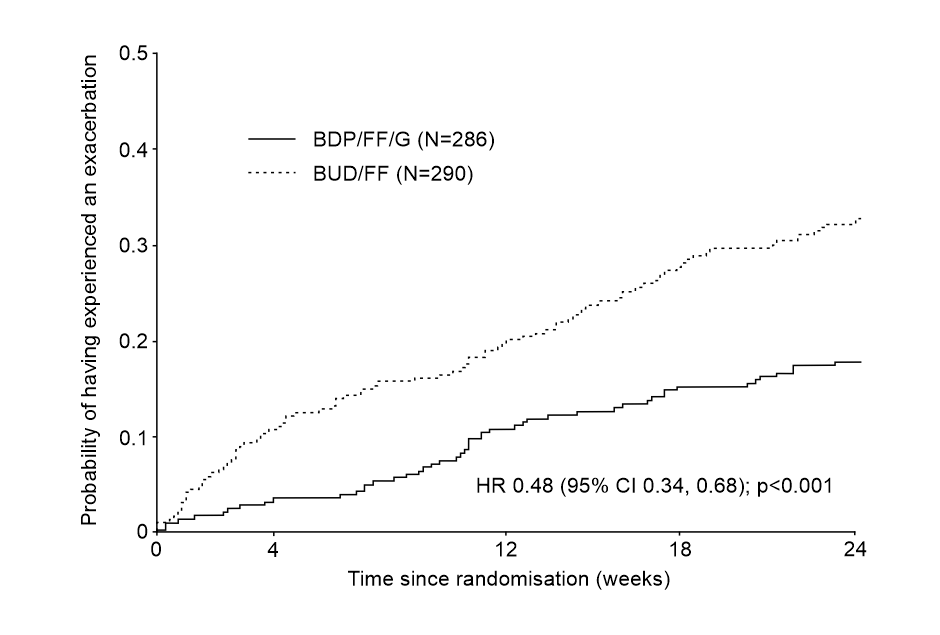


**B)**

The N values are for the ITT population. Abbreviations: COPD, chronic obstructive pulmonary disease; ITT, intention-to-treat; BDP, beclometasone dipropionate; FF, formoterol fumarate; G, glycopyrronium; BUD, budesonide; CI, confidence interval.

Supplementary Figure 8. Adjusted mean change from baseline in A) SGRQ and B) CAT total scores (China subgroup, ITT)


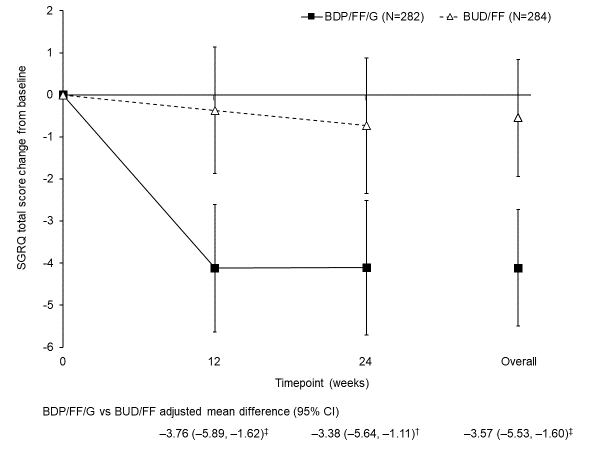


**A)**


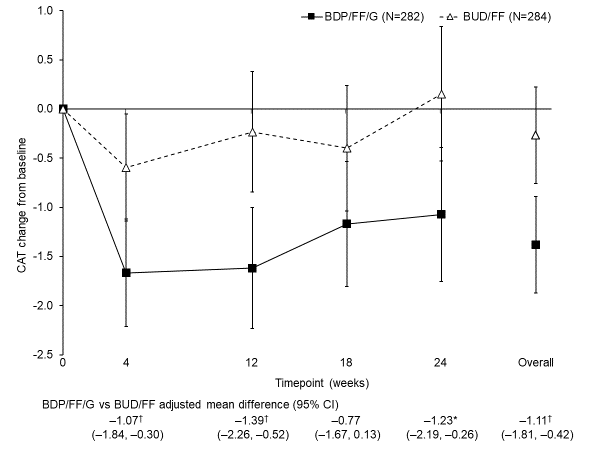


**B)**

*p<0.05, ^†^p<0.01, ^‡^p<0.001. Data are adjusted mean and 95% CI. The N values are the number of patients included in the statistical model. Abbreviations: SGRQ, St George’s Respiratory Questionnaire; CAT, COPD Assessment Test; ITT, intention-to-treat; BDP, beclometasone dipropionate; FF, formoterol fumarate; G, glycopyrronium; BUD, budesonide; CI, confidence interval.

Supplementary Figure 9. Adjusted mean change from baseline in percentage of days without rescue medication use, in A) the overall population and B) the China subgroup (ITT)

**A)**


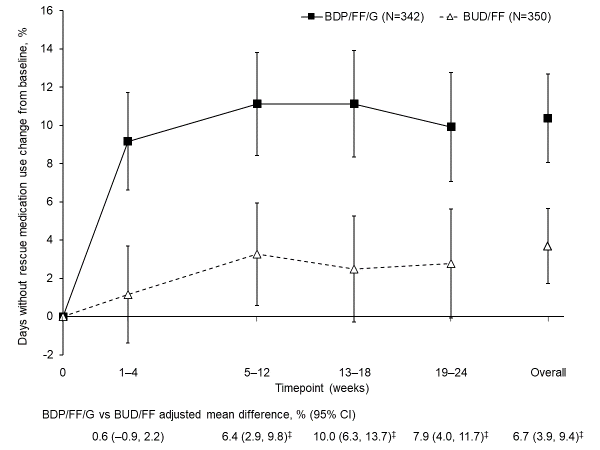


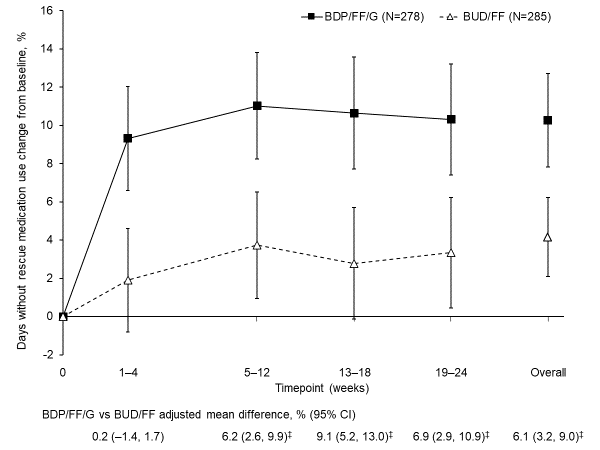


**B)**

^‡^p<0.001. Data are adjusted mean and 95% CI. The N values are the number of patients included in the statistical model. Abbreviations: ITT, intention-to-treat; BDP, beclometasone dipropionate; FF, formoterol fumarate; G, glycopyrronium; BUD, budesonide; CI, confidence interval.

Supplementary Figure 10. Adjusted mean change from baseline in average rescue medication use, in A) the overall population and B) the China subgroup (ITT)


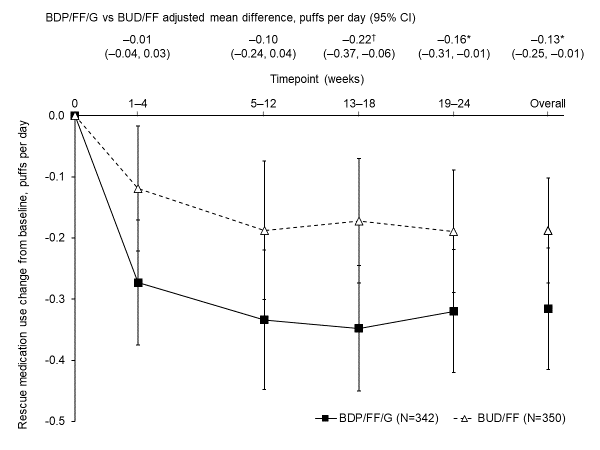


**A)**


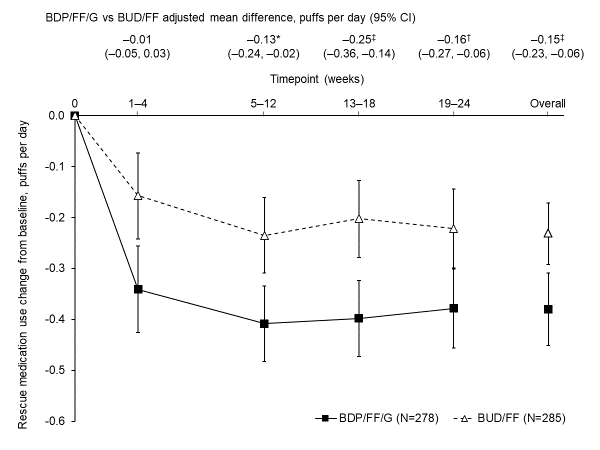


**B)**

*p<0.05, ^†^p<0.01, ^‡^p<0.001. Data are adjusted mean and 95% CI. The N values are the number of patients included in the statistical model. Abbreviations: ITT, intention-to-treat; BDP, beclometasone dipropionate; FF, formoterol fumarate; G, glycopyrronium; BUD, budesonide; CI, confidence interval.
